# Prediction of Oncogenic Interactions and Cancer-Related Signaling Networks Based on Network Topology

**DOI:** 10.1371/journal.pone.0077521

**Published:** 2013-10-25

**Authors:** Marcio Luis Acencio, Luiz Augusto Bovolenta, Esther Camilo, Ney Lemke

**Affiliations:** Department of Physics and Biophysics, Botucatu Biosciences Institute, UNESP – Univ Estadual Paulista, Botucatu, São Paulo, Brazil; University of Erlangen-Nuremberg, Germany

## Abstract

Cancer has been increasingly recognized as a systems biology disease since many investigators have demonstrated that this malignant phenotype emerges from abnormal protein-protein, regulatory and metabolic interactions induced by simultaneous structural and regulatory changes in multiple genes and pathways. Therefore, the identification of oncogenic interactions and cancer-related signaling networks is crucial for better understanding cancer. As experimental techniques for determining such interactions and signaling networks are labor-intensive and time-consuming, the development of a computational approach capable to accomplish this task would be of great value. For this purpose, we present here a novel computational approach based on network topology and machine learning capable to predict oncogenic interactions and extract relevant cancer-related signaling subnetworks from an integrated network of human genes interactions (*INHGI*). This approach, called *graph2sig*, is twofold: first, it assigns oncogenic scores to all interactions in the *INHGI* and then these oncogenic scores are used as edge weights to extract oncogenic signaling subnetworks from *INHGI*. Regarding the prediction of oncogenic interactions, we showed that *graph2sig* is able to recover 89% of known oncogenic interactions with a precision of 77%. Moreover, the interactions that received high oncogenic scores are enriched in genes for which mutations have been causally implicated in cancer. We also demonstrated that *graph2sig* is potentially useful in extracting oncogenic signaling subnetworks: more than 80% of constructed subnetworks contain more than 50% of original interactions in their corresponding oncogenic linear pathways present in the KEGG PATHWAY database. In addition, the potential oncogenic signaling subnetworks discovered by *graph2sig* are supported by experimental evidence. Taken together, these results suggest that *graph2sig* can be a useful tool for investigators involved in cancer research interested in detecting signaling networks most prone to contribute with the emergence of malignant phenotype.

## Introduction

The cancer phenotype is driven by the simultaneous expression of six biological capabilities: self-sufficiency in growth signals, insensitivity to antigrowth signals, avoidance of apoptosis, sustained angiogenesis, limitless replicative potential and tissue invasion and metastasis [1]. All these “hallmarks of cancer” emerge as a result of the complex interplay among oncogenic signals that are sets of sequential physical and biochemical reactions, i.e. phosphorylation, dephosphorylation, binding, dissociation etc., that are triggered by oncogenes or tumor suppressor genes and culminate in the expression of fundamental cell physiology changes associated with the malignant phenotype.

In general, oncogenic signals disturb the normal interactions as long as these signals propagate through the signaling network. For example, the overexpression of *CCND1*, a gene that is an important regulator in cell cycle progression, is the result of the constitutive oncogenic signaling triggered by mutated KRAS in many cancer cells [2]. The interactions downstream to KRAS and upstream to CCND1 are disturbed and, as a consequence, *CCND1* is overexpressed. However, overexpression of CCND1 alone is not sufficient to drive oncogenic transformation through the self-sufficiency in growth signals supported by mutated KRAS. Instead, additional oncogenic signals altering nuclear trafficking and ubiquitin-mediated proteolysis are required to promote the nuclear retention of the overexpressed CCND1 [3], condition of which the continued proliferation of cell, one of the features necessary to a full malignant transformation, can be sustained.

The above-mentioned example reinforces the fact that a normal cell will be transformed into a cancer cell only if multiple normal interactions are simultaneously disturbed by multiple oncogenic signals. In this regard, the determination of the oncogenic role of individual genes or proteins is insufficient to decipher the intricacies of the signaling pathways involved in cancer. The determination of oncogenic role of genes and proteins in a systems level, on the other hand, would be preferable to this end and, as a matter of fact, systems biology-based approaches have been convincingly shown to be successful in uncovering the functioning of cancer signaling pathways (for reviews on cancer systems biology, see [4] and [5]).

The combination of machine learning and graph theory is one of the systems biology-based approaches used to determine and predict how phenotypes emerge from the interactions among biological entities. We have previously used this approach to predict essential genes on a genome-wide scale and determine cellular rules for essentiality on *Escherichia coli* [6] and *Saccharomyces cerevisiae* [7]. Moreover, we have also used the combination of machine learning and graph theory to predict morbid and druggable genes and determine rules for morbidity and druggability in human [8]. Besides attaining successful prediction rates, we have also obtained biologically plausible cellular rules in these cases. These findings prompted us to investigate whether the combination of machine learning and graph theory would be also useful to reveal in a systems-level how cancer signaling pathways act in concert to generate the malignant phenotype.

For this purpose, we present in this paper a novel computational method based on machine learning and graph theory, the *graph2sig*, that determines (1) the oncogenic potential of an interaction, i.e. its capacity to transmit oncogenic signals in an integrated network of human gene interactions (INHGI) and (2) extracts from INHGI potential cancer-related signaling subnetworks given two genes of interest by using the oncogenic potential scores assigned to the interactions. Using *graph2sig*, we were able to reliably predict the oncogenic potential of interactions as well as to extract from *INHGI* subnetworks containing known and potential oncogenic pathways supported by experimental evidence. To the best of our knowledge, this is the first time that the combination of machine learning and graph theory is used to predict both the oncogenic potential of interactions and potential cancer-related signaling subnetworks.

## Materials and Methods

**Figure 1:**
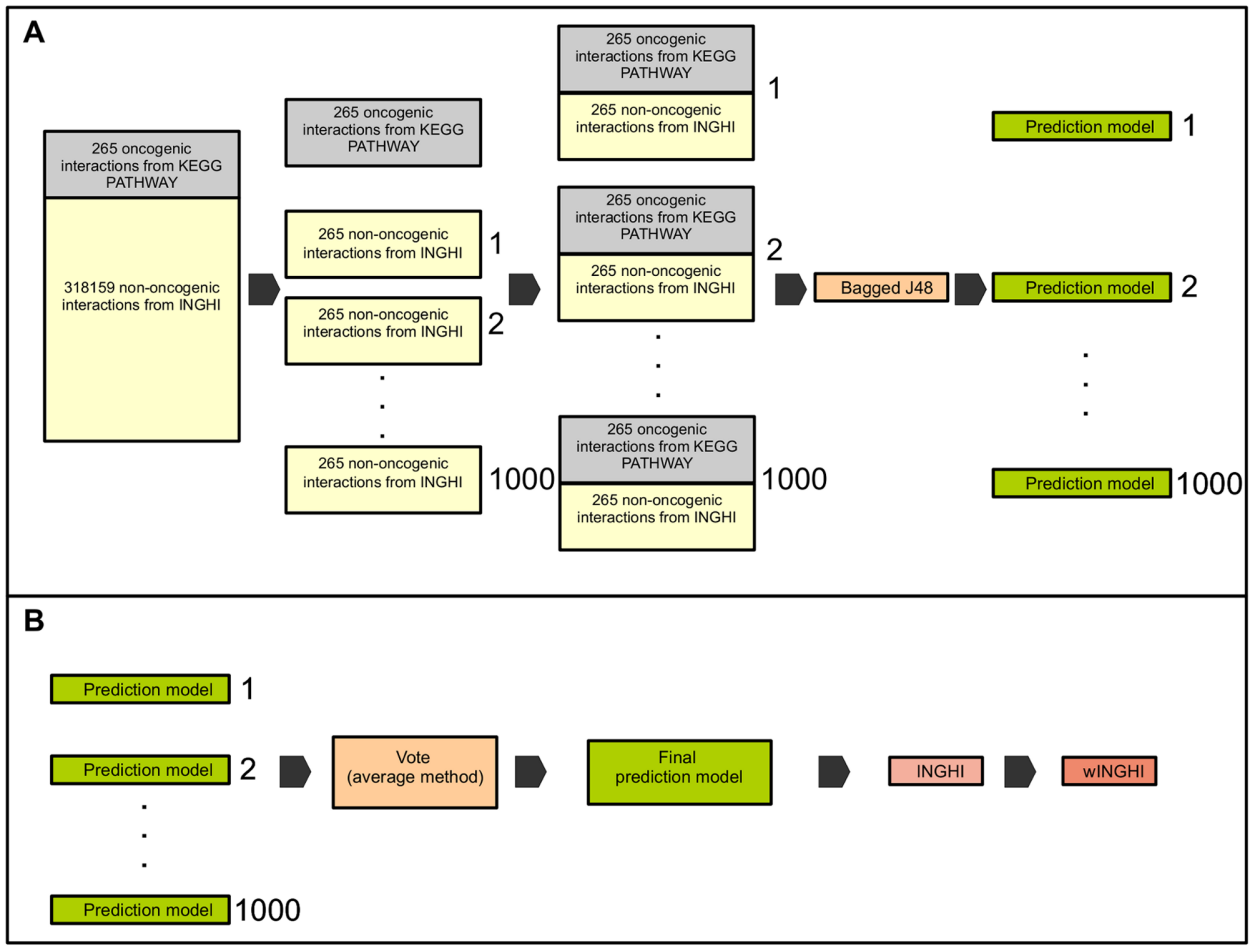
Initial steps of *graph2sig*. After building the *INHGI* and calculating the network centralities, balanced training groups are constructed and presented to the selected machine learning algorithm (bagged J48) that, in turn, generates the prediction models as depicted in **(A)**. These prediction models are combined in one final prediction model by the Vote algorithm. This final model is then used to assign oncogenic scores to interactions in *INHGI* originating the *wINHGI* as shown in **(B)**.

**Figure 2:**
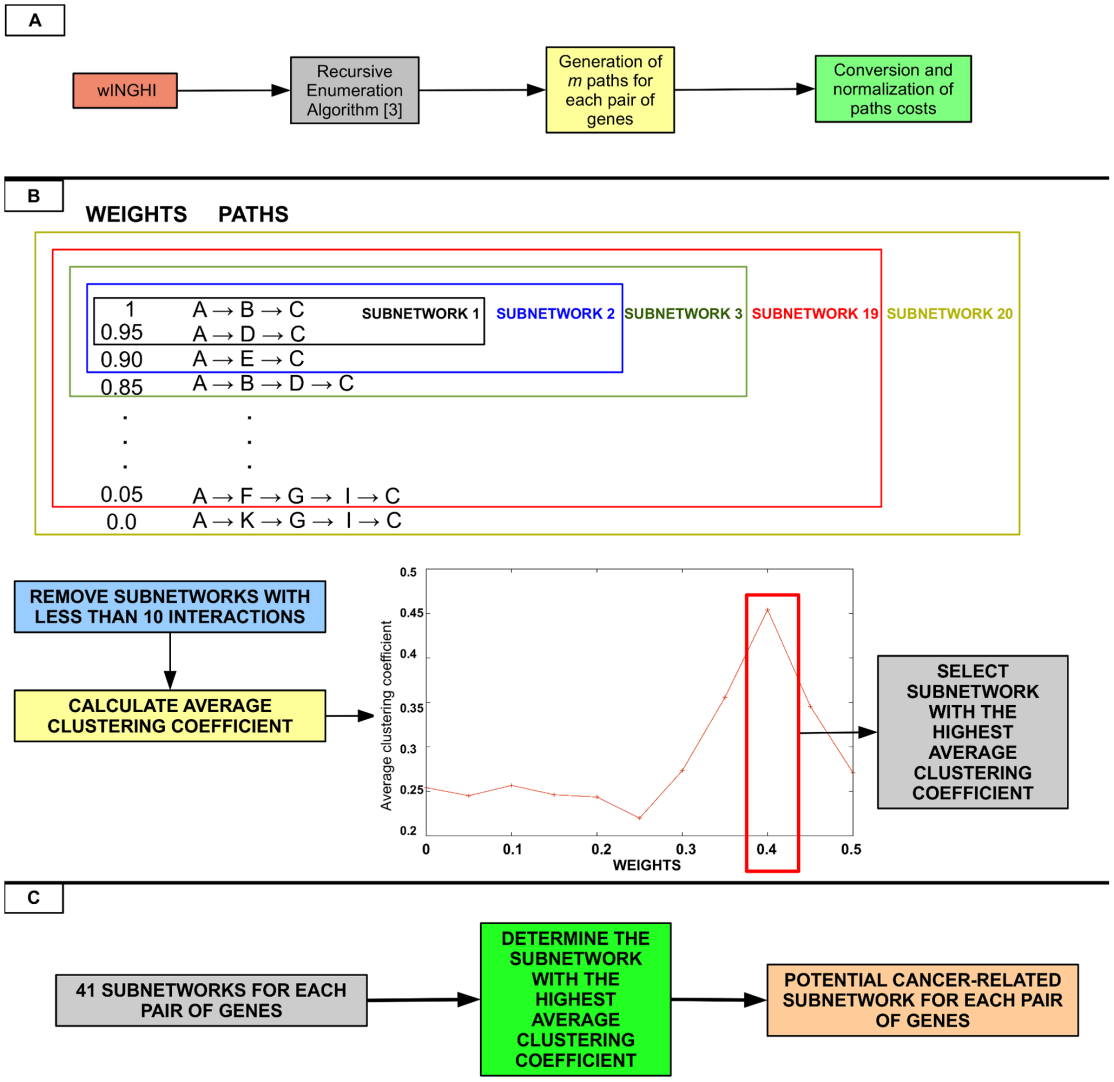
Final steps of *graph2sig*. (**A**) The application of *REA* on the *wINHGI* generates a list of 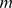 paths along with their costs for each pair of genes and these costs are converted to weights and normalized so that the minimum weight is zero and the maximum weight is 1. (**B**) Twenty subnetworks are generated from this list of paths and the subnetwork with the highest average clustering coefficient is selected. (**C**) For each pair of genes, 41 subnetworks are generated and, among these subnetworks, the one with the highest average clustering coefficient is selected as the final potential cancer-related subnetwork.

The aims of *graph2sig* is twofold: prediction of the oncogenic potential of interactions (Figure 1) and extraction of potential oncogenic signaling subnetworks from the *INHGI* (Figure 2). The first step of *graph2sig* is the construction of the *INHGI* and the computation of network centralities of genes in *INHGI* (Table 1). The second step concerns the use of these computed network centralities as training data for training machine learning algorithms (or learners) to generate prediction models for assigning oncogenic potential to interactions. The third step is the assignment of a “oncogenic potential” (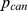) to each interaction by these prediction models (Figure 1).

**Table 1:** Network centralities measures used as training features in *graph2sig*.

Centrality measure	Function	Description
Degree centrality	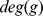	Number of links to gene 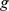 representing the number of interactions.
Clustering coefficient	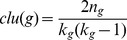	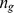 is the number of links connecting the neighbors of 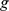 and 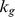 is the number of links connecting 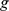 to its neighbors.
Betweenness centrality	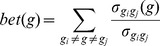	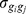 is the number of shortest paths between 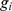 and 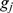 and 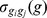 is the number of shortest paths between 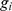 and 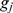 passing through 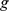 .
Closeness centrality	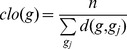	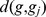 is the shortest distance between genes 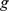 and 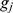 ; 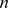 is the number of genes in the network.

The fourth step is to find the paths between two genes of interest, 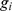 and 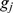, in the *INHGI* with the highest 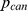 values by using the recursive enumeration algorithm (*REA*) [9], a path finding algorithm that lists the paths in the order of their weights (in this case, the 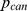). The final step is the selection and merging of paths found by *REA* for building the potential cancer-related signaling subnetwork containing the highest oncogenic pathways linking 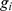 and 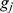 (Figure 2). These steps were implemented in a bash script available at http://www.lbbc.ibb.unesp.br/graph2sig.

### First step: *INHGI* construction and computation of network centralities

#### *INHGI* construction

The *INHGI*, which contains only experimentally verified interactions, was constructed based on assumption that two genes, 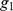 and 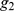, coding respectively for proteins 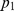 and 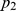, are interacting genes if *(i)* 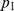 and 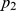 interact physically (protein physical interaction), *(ii)* the transcription factor 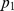 directly regulates the transcription of gene 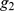, i.e., 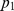 binds to the promoter region of 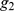 (transcriptional regulation interaction), or *(iii)* the enzymes 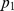 and 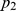 share metabolites, i.e., a product generated by a reaction catalyzed by enzyme 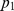 is used as reactant by a reaction catalyzed by enzyme 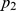, or the enzyme 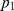 generates a metabolite that interacts with a non-enzymatic 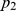 (metabolic interaction). The experimentally verified human interactions were obtained from different sources according to the type of interaction as described below.

Protein-protein physical interactions data were obtained from version 1.3 of the Human Integrated Protein-Protein Interaction rEference (HIPPIE), a database dedicated to the collection of experimentally verified and scored human protein-protein interactions integrated from multiple sources [10]. We collected from HIPPIE only interactions detected by experimental techniques that received scores of 5 or more, i.e. techniques that were considered by HIPPIE expert curators as those with high reliability and low error rate [10]. Protein-protein interactions from HIPPIE (and from all other similar databases in fact) are considered undirected interactions because this type of interaction is supposed to be non-directional. However, as the extraction of potential oncogenic signaling subnetworks from *INHGI* depends on the directionality of interactions, i.e. direction of signal flow between proteins, and interactions provided by our source of training data, the KEGG PATHWAY [11], are directed (see more details in the section “Construction of training datasets”), each protein-protein interaction 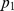 – 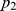 was transformed in two distinct directed interactions: 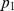 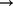 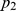 and 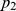 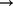 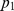.

Human transcriptional regulation interactions were obtained from the current version of the Human Transcriptional Regulation Interaction database (HTRIdb; [12]). Created by our group, HTRIdb is a repository of experimentally verified interactions between human transcription factors and their target genes detected by 14 distinct experimental techniques embracing both small and large-scale techniques. We collected from HTRIdb all transcription factors/target genes interactions.

Metabolic interactions were extracted from the human metabolic model Recon 1 [13] by a code implemented in Mathematica ^®^ 7.0 (Wolfram Research, Inc.). We excluded those metabolic interactions generated by the so-called “currency metabolites”, abundant molecular species present throughout the cell most of the time and, therefore, unlikely to impose any constraints on the dynamics of metabolic reactions [14]. We considered currency metabolites the eight most connected metabolites (ADP, ATP, H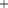, H
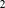
O, NADP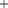, NADPH, orthophosphate and pyrophosphate) in the original metabolic model Recon 1. In addition, we added to the set of metabolic interactions some important interactions that are missing in the Recon 1: PIK3CA 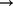 PDPK1, PIK3CA 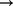 ILK, PIK3CA 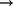 AKT3, PIK3CA 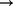 AKT2, PIK3CA 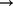 AKT1, PIK3CB 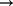 PDPK1, PIK3CB 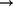 ILK, PIK3CB 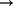 AKT3, PIK3CB 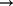 AKT2, PIK3CB 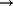 AKT1, PIK3CD 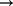 PDPK1, PIK3CD 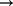 ILK, PIK3CD 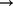 AKT3, PIK3CD 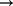 AKT2, PIK3CD 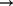 AKT1 and PTEN 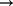 AKT1.

The final *INHGI* is a directed network formed by the integration of the protein physical, metabolic and transcriptional regulation interactions through genes common to these data sets (see Dataset S1). Before performing the integration, we converted all human gene names to their GeneID – as provided by the Entrez Gene database [15] – to avoid the creation of false interactions due to gene name ambiguity.

#### Computation of network centralities

For each gene 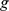 in *INHGI*, we computed 4 network centrality measures as listed in Table 1. Briefly, degree centrality (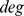) is defined as the number of links to node (in our case, gene). Clustering coefficient (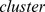) of a node (in our case, a gene) quantifies how close the node and its neighbors are to being a clique, i.e., all nodes connected to all nodes. For the *INHGI*, 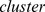 is defined as the proportion of links between the genes within the neighborhood of 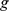 divided by the number of links that could exist between them. Betweenness centrality (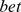) reflects the role played by a node (in our case, a gene) in the global network architecture and, for the *INHGI*, is defined as the fraction of shortest paths between 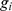 and 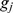 passing through 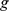. Closeness centrality (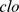) measures how close a node (in our case, a gene) is to all others in the network and, for the *INHGI*, is defined as the mean shortest path between 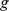 and all other genes reachable from it. All these network centrality measures were calculated by the Python package *NetworkX* 1.6 [16].

### Second step: generation of prediction models

#### Construction of training datasets

We constructed two groups of balanced training datasets, i.e., datasets containing the same number of positive (in our case, known oncogenic interactions) and negative (in our case, non-oncogenic interactions) examples: “normal datasets” and “shuffled datasets”. These training data are available at http://www.lbbc.ibb.unesp.br/graph2sig.

For constructing the training datasets, we first gathered a list of oncogenic interactions – interactions known to transmit oncogenic signals – from the cancer pathway maps provided by KEGG PATHWAY database [11] and then mapped them to the INHGI. The final list of oncogenic interactions used as positive examples to train our machine learning algorithm is comprised by 265 oncogenic interactions present in the INHGI (see Dataset S1). Regarding the negative examples, we considered as “non-oncogenic interactions” the remaining interactions present in the INHGI because currently it is not possible to build a list of interactions not known to transmit oncogenic signals. We randomly selected 1000 different sets of 265 of these non-oncogenic interactions and combine them with the list of 265 known oncogenic interactions to build 1000 different training datasets containing 530 interactions each. These are the “normal datasets”. From these normal datasets, we generate 10000 different “shuffled datasets” by randomly shuffling the class labels (oncogenic and non-oncogenic) among interactions (Figure 1).

#### Construction of prediction models

We employed the version 3.7.5 of WEKA (Waikato Environment for Knowledge Analysis) software package, a collection of machine learning algorithms for data mining tasks [17], to generate the prediction models. We used the training data described in the previous section to train the bootstrap aggregating (bagging), a machine learning ensemble meta-algorithm that combine multiple base learners [18]. In our case, we selected as the base learner the J48 algorithm, a WEKA's implementation of the C4.5 decision tree [19], with the default parameters.

Usually, the generation of prediction models by bagging is conducted as follows: (1) 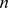 bootstrap replicates of the training dataset is created; (2) each replicate is presented to the base learner that than builds 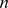 prediction models; and (3) these 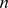 prediction models are eventually combined in a single model. In our case, bagging was configured to produce 20 bootstrap replicates of each training dataset and these replicates were then presented to J48 that, in turn, generated 20 prediction models for each training dataset. These models were finally combined in a single model for each training dataset totaling 1000 combined “normal” models (generated from the normal datasets) and 10000 combined “shuffled” models (generated from shuffled datasets).

#### Performance of constructed prediction models

We assessed the performance of our prediction models by estimating their recall, precision and area under the receiving operating characteristic (ROC) curve (AUC). Recall is the proportion of actual oncogenic interactions which are correctly predicted as such against all actual cancer-related interactions:
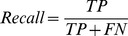



TP (true positive) denotes the amount of actual cancer-related interactions correctly predicted as such and FN (false negative) denotes the amount of actual cancer-related interactions incorrectly predicted as not known to be related to cancer, respectively.

Precision is the proportion of actual cancer-related interactions which are correctly predicted as such against all interactions predicted as related to cancer:
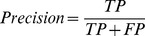



FP denotes the amount of interactions actually not known to be related to cancer incorrectly predicted as cancer-related interactions, respectively.

The AUC is a summary measure of the ROC curve – a plot of the true positive rate versus false positive rate that indicates the probability of a true positive prediction as a function of the probability of a false positive prediction for all possible threshold values [20] – and is equivalent to the probability that a randomly chosen negative example (in our case, a non-oncogenic interaction) will have a smaller estimated probability of belonging to the positive class than a randomly chosen positive example (in our case, a oncogenic interaction) [21].

Using WEKA, we estimated the above-mentioned performance measures by performing a 10-fold cross-validation to test the 1000 combined normal and 10000 combined shuffled prediction models. The 10-fold cross-validation works as follows: each dataset is randomly partitioned into 10 subsets. Of the 10 subsets, a single subset is retained as the validation data for testing the model, and the remaining 9 subsets are used as training data. The cross-validation process is then repeated 10 times, with each of the 10 subsets used exactly once as the validation data. The 10 results from the folds are then averaged to produce a single estimation for each performance measure for each prediction model. In our case, each performance measure of each prediction model is an average of 200 results since each model is a combination of 20 other models. Finally, we reported the performance measures estimated by the 10-fold cross-validation as medians of the 1000 combined normal and 10000 combined shuffled prediction models.

The statistical comparisons of the performance measures estimated by our prediction models generated by normal and shuffled datasets were performed by the Mann-Whitney-U test [22]. According to established conventions in the machine learning community, we used this test since it makes no assumptions about the underlying distribution of performance measures used to evaluate the prediction models [23]. Differences between performance measures estimated by our prediction models generated by normal and shuffled datasets with a p-value 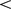
0.005 were considered statistically significant.

### Third step: prediction of potential oncogenic interactions

We assembled the 1000 combined normal prediction models constructed in the previous step in one single model (available at http://www.lbbc.ibb.unesp.br/graph2sig) by using “Vote”, a WEKA's implementation of the voting meta-algorithm that combines the output predictions of each prediction model by different rules [24]. We then applied this single prediction model, which contains 20000 models as a result of the combination of the 1000 combined models that, in turn, contains 20 models each, to assign 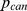 values, i.e., potential to transmit oncogenic signals, to the entire set of interactions in INHGI 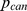 values. The final 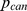 value is an average of 20000 values individually assigned by each model within the single prediction model.

### Fourth step: execution of the recursive enumeration algorithm (*REA*)

To find the paths with the highest 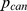 values between two genes 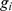 and 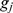 in the *INHGI*, *graph2sig* uses *REA* [9]. This algorithm enumerates 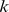 paths between a start and an end node in the reverse order of their costs, 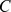, so that the path with minimum 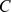 is ranked first among the 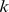 paths. Before executing *REA*, 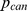 values in *INHGI* are converted into costs (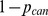) since *REA* considers the weights of edges as costs. In this way, the path with the maximum 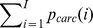, where 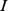 is the total number of interactions in the path, corresponds to the path with minimum 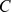 for *REA*.

In *REA*, besides selecting a start node – in our case a gene 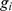 that triggers the oncogenic signal – and an end node – in our case a gene 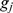 of interest that receives the oncogenic signal triggered by the start gene – it is also possible to define 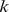 up to a maximum value predetermined for each size of network. For *INHGI*, for instance, *REA* allows to define a maximum 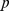 of 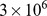 paths. For each pair 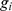 – 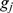, *graph2sig* runs *REA* with 41 different values of 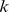: 100 to 1000 in increments of 100 paths, 2000 to 10000 in increments of 1000 paths, 20000 to 100000 in increments of 10000 paths, 200000 to 1000000 in increments of 100000 paths and 1500000 to 3000000 in increments of 500000 paths.

From the 41 groups of paths returned by *REA*, 41 potential cancer-related signaling subnetworks are constructed for each 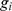 – 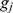 pair as shown in the next section.

### Final step: extraction of potential cancer-related signaling subnetworks

In this final step of *graph2sig*, from each group of paths returned by *REA* (e.g., group with 1000 paths or 100000 paths) for each 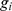 – 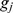 pair, the potential cancer-related signaling subnetwork is constructed as follows:

1. For each path, 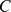 is converted to weight, 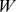, where 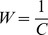;

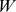
 values are normalized so that 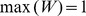 and 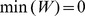 as following:
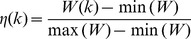
(1)where 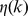 is the normalized 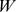 for path 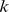 and 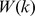 is the calculated weight in (1) for path 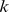;
3. Twenty subnetworks are constructed such that each subnetwork is comprised by a set of 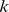 paths with 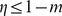 where 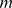 ranges from 0 to 0.95 in increments of 0.05 (Figure 2);
4. The subnetwork with the highest average clustering coefficient among all 20 subnetworks is selected as the potential cancer-related signaling subnetwork (Figure 2).

At this level, *graph2sig* contains a collection of 41 potential cancer-related signaling subnetworks for each 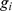 – 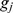 pair. The ultimate potential cancer-related signaling subnetwork for each 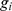 – 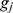 pair is the subnetwork with the highest average clustering coefficient among the 41 subnetworks (Figure 2).

## Results and Discussion

### *INHGI*: general features

The construction of the *INHGI* is fundamental to *graph2sig* since the utilization of network centrality measures of genes as training features in the machine learning approach proposed here is the core of the whole process. In addition, the extraction of a signaling subnetwork makes sense only in a network context. Thus, it is important to be aware of some general features of the *INHGI* as these features can serve as useful resources for the analysis and interpretation of results.

The *INHGI* is a directed network comprised by 19789 genes and 318332 interactions. From these 19789 genes, 13932 interact with each other via 242716 protein physical interactions (considered here as directed interactions; see details in “Methods”), 1166 via 24299 metabolic interactions and 18310 via 51317 transcriptional regulation interactions. Moreover, 896 genes interact with each other via protein physical and metabolic interactions, 12508 via protein physical and transcriptional regulation interactions and 1042 via metabolic and transcriptional regulation interactions (see Dataset S1).

The *INHGI* is certainly far from complete if we consider, for example, the estimates calculated by Stumpf and colleagues [25]: they have estimated that the size of human network of protein-protein interactions is about 650000 interactions. Therefore, *INHGI* contains 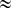
19% of total number of estimated human protein-protein interactions as 121358 undirected protein-protein interactions are present in this network. Moreover, *INHGI* contains approximately 46% of the already identified 43059 human genes (according to the EntrezGene database [15] accessed on September 10th, 2012). The remaining 23211 genes absent from *INHGI* are transcriptionally regulated by at least one transcription factor implying that, in the future, *INHGI* will be increased by the addition of at least 23211 transcriptional regulation interactions.

Due to the incompleteness of the *INHGI* discussed above – in fact a noticeable characteristic of all networks constructed exclusively by experimentally validated interactions –, the results described in the next sections are valid only for the current *INHGI*. Any alteration in the structure of *INHGI* will also change the network centrality measures and, as a consequence, the construction of prediction models as well as the assignment of 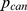 values.

### Evaluation of the performance of prediction models

The second and third steps of *graph2sig* concern, respectively, the generation of prediction models and assignment of oncogenic potential scores, 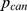, to interactions in *INHGI*. Prior to the assignment of 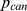 values (as described in detail in “Methods”), we sought to estimate the performance of the generated prediction models in recovering known oncogenic interactions and distinguishing non-oncogenic from oncogenic interactions. For this purpose, we assessed their performance by measuring their median recall, precision and AUC across the 1000 normal models (see “Methods” for more details).

Before analyzing the performance measures of our prediction models, we estimated the performance measures of the prediction models generated from the shuffled datasets and then compared them with the prediction models generated from the normal datasets. This was done to check whether the prediction models built by training the bagged J48 on non-shuffled datasets learned the traits actually associated with cancer instead of traits associated with any random subset of genes. For this comparison, we used the Mann-Whitney-U test [22] as described in “Methods”. For shuffled models, the recall ranged from 0.22 to 0.81 with a median of 0.49, the precision ranged from 0.39 to 0.69 with a median of 0.5 and the AUC ranged from 0.38 to 0.62 with a median of 0.49. All these values are statistically different from the performance measures of normal models (p-value 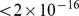 for all measures), thereby indicating that the traits actually associated with cancer were learned by our normal prediction models.

**Figure 3:**
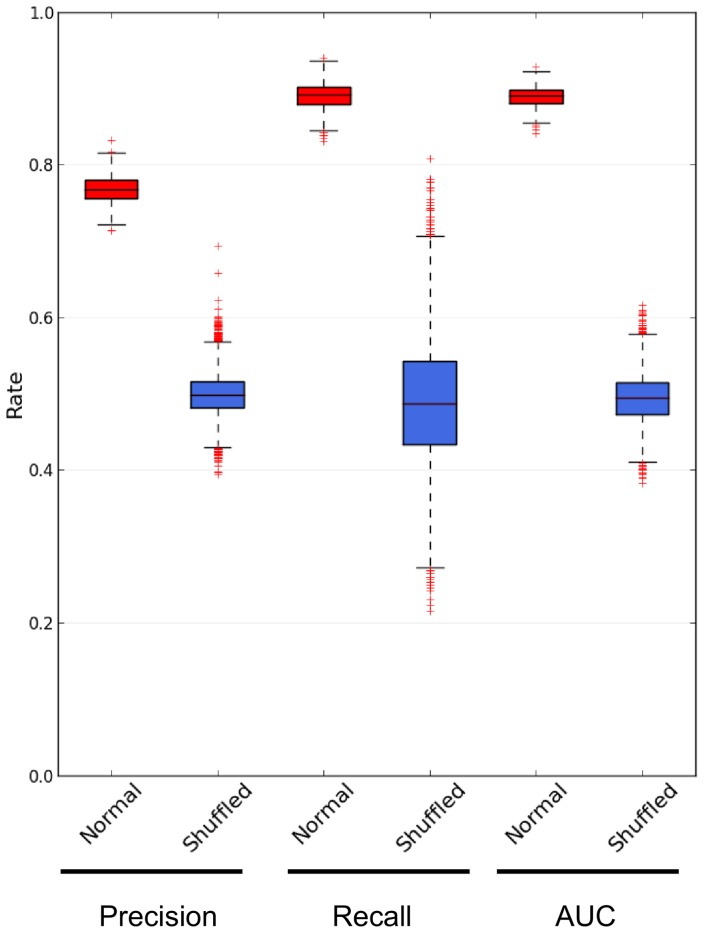
Boxplot showing the predictive performance measures for prediction models. Boxplot showing the distribution of recall, precision and AUC values for 1000 prediction models generated from normal datasets (red boxes) and 10000 prediction models generated from shuffled datasets (blue boxes). The distributions of performance values for models generated from normal and shuffled datasets are statistically different according to the Mann-Whitney-U test (p-value 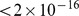 for all measures).

After confirmation that the prediction models generated from normal datasets is likely to learn the traits actually associated with cancer, we aimed to analyze their performance measures. As shown in Figure 3, the recall of prediction models ranged from 0.83 to 0.94 with a median of 0.89 and their precision ranged from 0.71 to 0.83 with a median of 0.77. Then, the prediction models correctly recovered 89% of known oncogenic interaction with a precision of 77%. Furthermore, the probability of an interaction predicted as oncogenic actually belongs to the set of known oncogenic interactions ranged from 84% to 93% with a median of 89% as indicated by the median AUC (Figure 3).

While our prediction models are able to recover most of known oncogenic interactions as revealed by their high recall (median of 89%), their ability to distinguish oncogenic from non-oncogenic is less pronounced as revealed by their moderate precision (median of 77%). This indicates a certain level of noise in the training data that is likely associated with the existence of shared common features between oncogenic and non-oncogenic interactions that induced our prediction models to yield a moderate performance in discriminating oncogenic from non-oncogenic interactions. This can be partially due to the strategy used to select non-oncogenic interactions: since it is impossible at present to compile a list of non-oncogenic interactions, we selected interactions not known to transmit oncogenic signals, i.e., all interactions in INHGI except the known oncogenic interactions, as non-oncogenic interactions. Thus, some of these non-oncogenic interactions may actually be existing oncogenic interactions not yet present in the cancer pathway maps provided by KEGG PATHWAY database.

Our strategy for selecting the oncogenic interactions could also have contributed to the existence of shared common features between oncogenic and non-oncogenic interaction. As previously mentioned in the section “Materials and Methods”, we considered as “oncogenic” those interactions present in the cancer pathways maps provided by KEGG PATHWAY database. We cannot guarantee the real “oncogenicity” of these interactions since these cancer pathways maps are inferred from literature by KEGG expert curators through the combination of experimental data obtained from different research articles. No experimental evidence has been reported so far to show that these pathways, at least in their entirety, are actually utilized in cancer cells. Although we did not try to construct training groups by using oncogenic interactions collected from sources other than KEGG PATHWAY, we believe that it is difficult to avoid this uncertainty about the real oncogenicity of interactions. For example, the pathways present both in the NetPath database [26] and in the oncogenic signaling map constructed by Cui and colleagues [27], two alternatives to KEGG PATHWAY for gathering oncogenic interactions, are also collected by using the same strategy as in the case of KEGG PATHWAY.

Another contributing factor for the existence of shared common features between non-oncogenic and oncogenic interactions can be the incompleteness of INHGI as previously discussed. Since our network contains about 121000 undirected protein-protein interactions in comparison to the estimated 650000 human protein-protein interactions [25], we can envisage that the values of all network centrality measures might change with the enlargement of network size and, therefore, some of the network centralities-related shared common features between oncogenic and non-oncogenic interactions might disappear as a consequence.

### Do 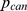 values reliably express the oncogenic nature of interactions?

As the final goal of *graph2sig* is to use the 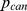 values as edge weights for the extraction of oncogenic signaling subnetworks between any two genes of interest in the *INHGI*, it is important to check whether these values reliably express the oncogenic nature of interactions. For this purpose, the prediction models evaluated in the previous section were merged in a single model that, in turn, was used to assign 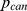 values to all interactions in the *INHGI* (see details in “Methods”). This weighted *INHGI* will be hereafter denoted by *wINHGI*.

**Figure 4:**
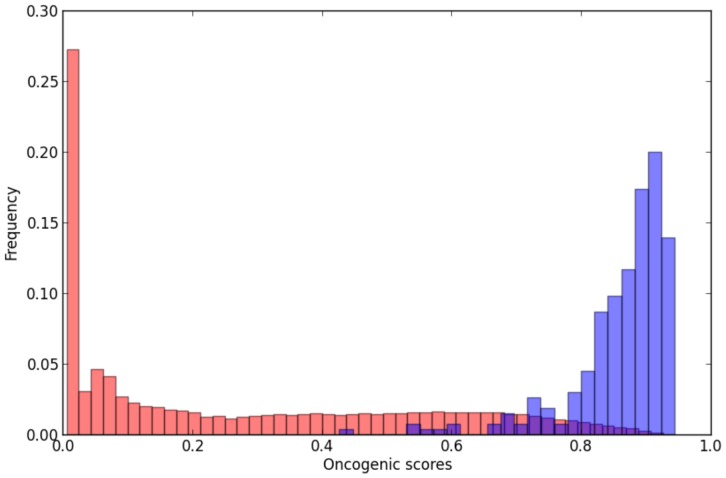
Frequency distribution of known oncogenic interactions per intervals of oncogenic scores. The blue and red bars show, respectively, the frequency distributions of known oncogenic interactions and all interactions in the *wINHGI* per 0.2 intervals of oncogenic scores.

Our prediction model seems indeed to express the oncogenic nature of interactions: known oncogenic interactions clearly received high oncogenic potential scores as shown by Figure 4. In fact, by using a hypergeometric test – statistical test that calculates the likelihood, in a p-value form, that the overrepresentation of a certain category in a sample occurs by chance – we showed that the 257 known oncogenic interactions, which represent 0.08% of interactions in *wINHGI* and 0.8% of the 30395 interactions that received 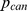 values greater than 0.7 (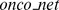), are significantly overrepresented in the 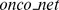 with a p-value 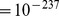. Furthermore, 252 (95%) of the 265 known oncogenic interactions were assigned values of 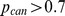.

The fact that known oncogenic interactions are overrepresented in 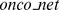 is not surprising since these interactions were used as training examples for constructing the prediction model. To convincingly demonstrate that 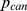 values reliably express the oncogenic nature of interactions, we asked whether putative oncogenic interactions – interactions that seem to be involved in cancer and are currently absent from the KEGG PATHWAY database – also received high oncogenic potential scores by our prediction model and are also significantly overrepresented in 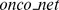. To achieve this goal, we considered as putative oncogenic interactions the “oncogenic signal transduction events” defined by Cui and colleagues [27]. According to these investigators, oncogenic signal transduction events are interactions in which the upstream and downstream nodes get altered either genetically or epigenetically and, therefore, they are most likely to be selected and used in cancer signaling. Our oncogenic transduction events are interactions in the *wINHGI* in which both genes are those for which mutations have been causally implicated in cancer. These genes were collected from the Cancer Gene Census (http://www.sanger.ac.uk/genetics/CGP/Census/; [28]).

**Figure 5:**
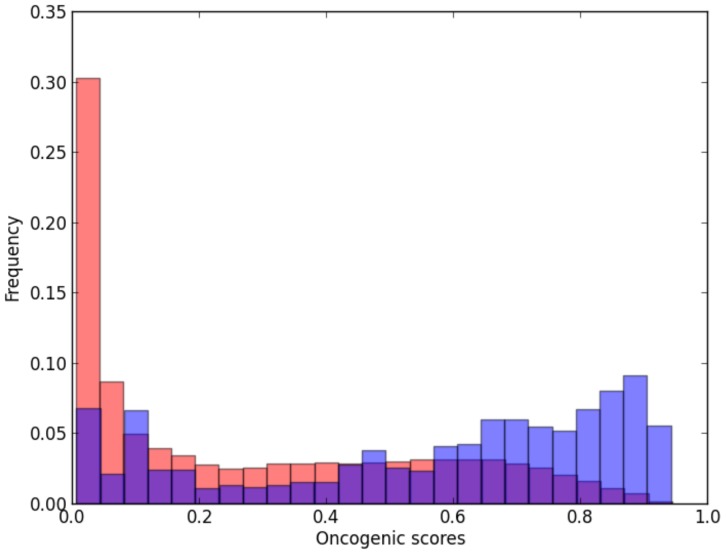
Frequency distribution of potential oncogenic interactions per intervals of oncogenic scores. The blue and red bars show, respectively, the frequency distributions of potential oncogenic interactions and all interactions in the *wINHGI* per 0.4 intervals of oncogenic scores.

As shown in Figure 5, our prediction model tended to assign high oncogenic potential scores to these oncogenic transduction events although this assignment is not as clear as in the case of known oncogenic interactions. However, by using a hypergeometric test, we showed that the oncogenic signal transduction events, which represent 0.3% of interactions in *wINHGI* and 1.5% of interactions in 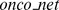, are significantly overrepresented in 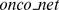 with a p-value 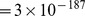. Moreover, 470 (43%) of the 1066 oncogenic signal transduction events in *wINHGI* were assigned values of 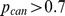.

### Determination of oncogenic signaling subnetworks in the *wINHGI*

As shown in the previous section, the oncogenic scores assigned by the first step of *graph2sig* seem indeed to reflect the oncogenic nature of interactions. However, as mentioned in “Introduction”, a normal cell will be transformed into a cancer cell only if multiple normal regulatory interactions are simultaneously disturbed by multiple oncogenic signals. This prompted us to proceed to the last steps of *graph2sig*: to use the oncogenic scores as edge weights in the extraction of oncogenic signaling subnetworks between any two genes of interest in the INHGI (Figure 2).

To evaluate the performance of *graph2sig* on extracting cancer signaling subnetworks between genes of interest, oncogenic linear pathways (OLPs) extracted from cancer pathway maps provided by KEGG PATHWAY database were checked for their presence within the extracted subnetworks. As currently there is no database dedicated to the collection of experimentally validated cancer signaling subnetworks, we were forced to use the OLPs as surrogates for assessing the performance of *graph2sig*. For this purpose, we selected OLPs from which all interactions could be mapped to INHGI and the initial gene was an oncogene or tumor suppressor gene. In addition, we selected OLPs from which the oncogenic signal triggered by the initial gene reaches the target genes only through direct interactions. Using this strategy, we obtained 52 OLPs with number of interactions ranging from 3 to 8 (Table S1). We then used *graph2sig* to extract from INHGI the cancer signaling subnetworks between the initial and target genes from each OLP.

From the 52 pairs of genes collected from the above-mentioned OLPs, *graph2sig* extracted subnetworks with size ranging from 10 to 3273 interactions (Table S1 and Dataset S2). Thirty-two subnetworks (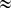
61% ) contain all interactions from their corresponding OLPs and 43 subnetworks (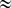
83%) contain 50% or more interactions from their corresponding OLPs (Table S1). Before proceeding to the analysis of subnetworks per se, we checked whether the success rate of *graph2sig*, i.e. the ratio between the number of interactions of the OLP in the subnetwork and the actual number of interactions in OLP, was dependent on factors other than the availability of pathways with high oncogenic scores linking the selected initial and target genes.

First, we examined the apparent dependence of success rate of *graph2sig* on OLP size. At a first glance, the success rate of *graph2sig* seems to rely on the OLP size: as we can observe in the Table S1, all subnetworks constructed from OLPs with 3 interactions and 80% of subnetworks constructed from OLPs with 4 interactions contain all interactions from their corresponding OLPs. On the other hand, only 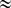
23% of the subnetworks constructed from OLPs with more than 4 interactions contain all interactions from their corresponding OLPs. To ascertain whether there is indeed a dependence between the success rate of *graph2sig* and OLP size, we applied the Kendall's rank correlation test to assess the correlation strength between these variables. According to this test, success rate of *graph2sig* and OLP size correlate moderately with each other (Kendall correlation coefficient 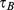 = −0.56, p-value  = 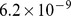). Therefore, the performance of *graph2sig* is not strongly influenced by OLP size.

Second, we attempted to determine whether the success rate of *graph2sig* could be dependent on the ratio between the sizes of the subnetwork and the corresponding OLP (subnet:OLP ratio). According to Table S1, 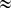
68% of subnetworks with subnet:OLP ratio greater than or equal to 10 contain all interactions of the corresponding OLPs while 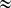
40% of subnetworks with subnet:OLP ratio less than 10 contain all interactions of the corresponding OLPs. We performed a Kendall's rank correlation test that showed a weak correlation (Kendall correlation coefficient 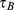 = 0.31, p-value  = 0.001) between the success rate of *graph2sig* and subnet:OLP ratio. Thus, the performance of *graph2sig* is also not strongly influenced by the subnet:OLP ratio.

It is worth to point out, however, that these correlations between the success rate of *graph2sig* and the OLP size and the subnet:OLP ratio as well as the success rate of *graph2sig* itself should be interpreted cautiously. As already discussed in the section “Evaluation of the performance of prediction models”, OLPs are pathways inferred from literature by KEGG expert curators through the combination of experimental data obtained from different research articles [29]. To the best of our knowledge, so far no experimental evidence has been reported to show that these OLPs, at least in their entirety, are actually utilized in cancer cells. This limitation regarding the usage of OLPs as references is thus likely to underestimate the performance of *graph2sig* due to the uncertainty about the real role of these OLPs in transmitting oncogenic signals as demonstrated in the maps provided by KEGG PATHWAY database. However, on the other hand, *graph2sig* can be evaluated by its performance in detecting known oncogenic pathways that are not currently visible in the KEGG PATHWAY maps. Below, we give some examples that illustrate this point.

**Figure 6:**
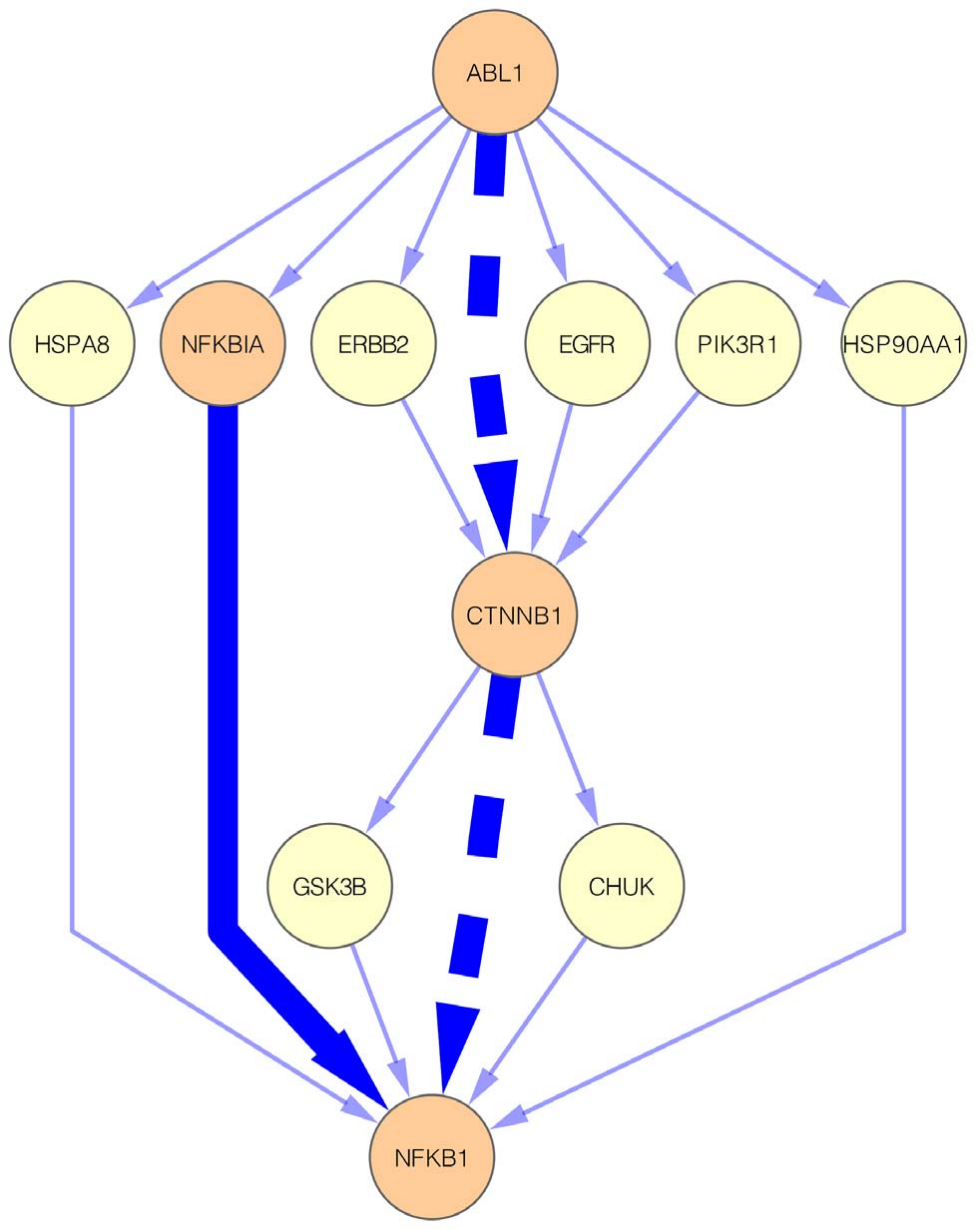
The ABL1 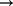 NFKB1 subnetwork. This subnetwork contains 18 interactions. The highlighted solid edge represents the interaction presents in the corresponding OLP. The highlighted dashed edges represent the interactions of the potential oncogenic pathway (ABL1 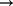 CTNNB1 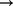 NFKB1). Blue edges represent protein physical interactions and orange nodes represent genes participating in the known or potential oncogenic pathways.

The ABL1 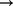 NFKB1 subnetwork contain 18 interactions and, among these interactions, only one, specifically the physical interaction between proteins NFKBIA and NFKB1, is also present in its corresponding OLP (Figure 6 and Dataset S2). Despite this, further analysis of the ABL1 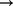 NFKB1 subnetwork revealed the presence of an oncogenic pathway not described in KEGG PATHWAY: ABL1 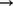 CTNNB1 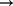 NFKB1 (Figure 6). The existence of this pathway in cancer cells is supported by experimental evidence reported in two research articles [30], [31]. The cancer-related ABL1/CTNNB1 interaction has been demonstrated by Colluccia and colleagues that showed that ABL1 phosphorylates CTNNB1 and this phosphorylation is responsible for stabilization and nuclear translocation of CTNNB1 in chronic myeloid leukemia [30]. The cancer-related CTNNB1/NFKB1 interaction, in turn, has been reported by Deng and colleagues that demonstrated that CTNNB1 interacts with and inhibits NFKB1 in human colon and breast cancers [31]. Therefore, *graph2sig* disclosed a potential pathway in which the activity of NFKB1 is disrupted by oncogenic signals received by ABL1 via CTNNB1.

The MET 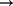 JUN subnetwork contain 116 interactions and, among these interactions, only two, specifically the protein physical interactions MET/GRB2 and MAPK1/JUN, are also present in its corresponding OLP (Figure S1). Despite this, further analysis of the MET 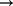 JUN subnetwork allowed us to find an oncogenic pathway absent from KEGG PATHWAY: MET 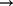 STAT3 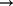 JUN (Figure S1). Experimental evidence supporting this pathway comes from two research articles [32], [33]. The oncogenic MET/STAT3 interaction has been detected by Syed and colleagues that demonstrated that the disruption of this interaction can block tumor cell invasion in an in vivo model [32]. The involvement of STAT3/JUN interaction in tumor progression has been demonstrated by Ivanov and colleagues [33]: they reported that a cooperation between STAT3 and JUN downregulates FAS surface expression and its downregulation underlies the resistance of melanoma and possibly other tumor types to therapy. Hence, by using *graph2sig*, we found a potential oncogenic pathway by which the oncogenic signals triggered by MET can reach JUN.

**Figure 7:**
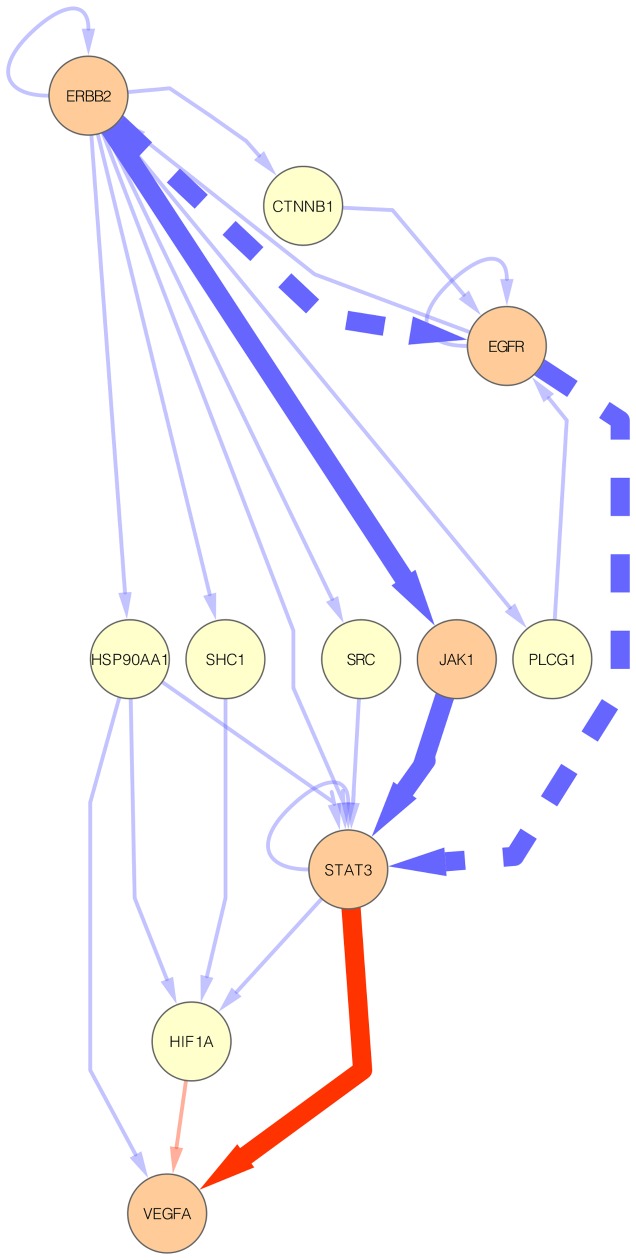
The ERBB2 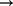 VEGFA subnetwork. This subnetwork contains 24 interactions. The highlighted solid edges represent the interactions present in the corresponding OLP. The highlighted dashed edges represent the interactions of the potential oncogenic pathway (ERBB2 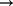 EGFR 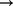 STAT3 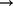 VEGFA). Blue and red edges represent, respectively, protein physical and transcriptional regulation interactions; orange nodes represent genes participating in the known or potential oncogenic pathways.

The ERBB2 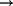 VEGFA subnetwork contains 24 interactions and, among these interactions, all three interactions of its corresponding OLP are also present in this subgraph (Figure 7 and Dataset S2). Regardless of the presence of a complete known oncogenic pathway, further analysis of the ERBB2 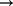 VEGFA subnetwork allowed us to find a potential oncogenic pathway absent from KEGG PATHWAY: ERBB2 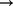 EGFR 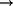 STAT3 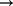 VEGFA (Figure 7). While the STAT3/VEGFA is a known oncogenic transcriptional regulation interaction present in KEGG PATHWAY, the other two interactions are oncogenic interactions supported by experimental evidence as shown by two research articles [34], [35]. The oncogenic ERBB2/EGFR interaction has been detected by Wang and colleagues that demonstrated that ERBB2 associates with and activates the EGFR in lung cancer cells [34]. The involvement of EGFR/STAT3 interaction in tumor progression, in turn, has been demonstrated by Jaganathan and colleagues [20]: they reported that the EGFR/STAT3 interaction supports the pancreatic cancer phenotype and explains in part the insensitivity of pancreatic cancer cells to the inhibition of EGFR or STAT3 alone. Thus, by using *graph2sig*, we found a potential oncogenic pathway by which the oncogenic signals triggered by ERBB2 alters the expression of VEGFA via EGFR-STAT3 interaction.

As a final example, we checked the KRAS 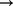 CCND1 subnetwork (Figure S2) for the presence of novel potential oncogenic pathways. This subnetwork contains 134 interactions including all five interactions of its corresponding OLP (Figure S2). The analysis of the KRAS 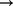 CCND1 subnetwork revealed a potential oncogenic pathway that is partially shown in KEGG PATHWAY: KRAS 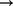 PIK3CA 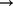 AKT1 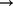 GSK3B 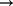 MYC 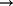 CCND1 (Figure S2). All interactions, except for GSK3B/MYC, can be found in other OLPs (e.g. in KRAS 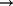 BCL2L1; see Dataset S2). The oncogenic role of GSK3B/MYC interaction, in turn, has been demonstrated elsewhere [36]. Therefore, we can hypothesize that, in cancer cells, the expression of CCND1 promoted by MYC can be a result of oncogenic signals triggered by KRAS that eventually protects MYC from degradation by GSK3B.

Taken together, these results, i.e. the high fraction (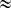 83%) of constructed subnetworks containing 50% or more interactions from their corresponding OLPs and the discovery of oncogenic pathways experimentally reported in literature, are compelling enough to suggest that the oncogenic scores assigned to interactions in the first step of *graph2sig* can reliably be used as edge weights in the extraction of oncogenic signaling subnetworks between any two genes of interest in the INHGI in the second step of *graph2sig*.

### Concluding remarks

In an effort to accelerate the pace of discovery of cancer-related interactions and subnetworks, we designed a network topology-based machine learning computational approach, the *graph2sig*, that uses network centralities as training attributes to construct prediction models capable to assign oncogenic scores to interactions that, in turn, are the base for the extraction of cancer-related signaling subnetworks.

We could demonstrate that the combination of machine learning and graph theory is promising in prioritizing (1) interactions capable to transmit oncogenic signals and (2) cancer-related signaling subnetworks. Similarly to the predictive performance of models constructed to predict essential genes in *Escherichia coli* [6] and *Saccharomyces cerevisiae* [7] and morbid and druggable genes in human [8], the prediction model constructed in the first steps of *graph2sig* presented a predictive performance reliable enough (median recall of 0.89, median precision of 0.77 and median AUC of 0.89) to assign oncogenic scores to the interactions of the INHGI. From this finding we can conclude that network centralities are predictive of the oncogenic nature of interactions.

Regarding the utilization of oncogenic scores as edges weights for the extraction of oncogenic signaling subnetworks, we reason that network centralities, indirectly through the oncogenic scores, are also predictive of cancer-related signaling subnetworks since more than 80% of constructed subnetworks contain more than 50% of original interactions in their corresponding OLPs. In addition, the novel potential oncogenic pathways originally absent from KEGG PATHWAY but embedded in the constructed oncogenic signaling subnetworks seem to be biologically plausible as demonstrated by experimental evidence taken from the biomedical literature.

To the best of our knowledge, this is the first time that the combination of machine learning and graph theory is used to predict both the oncogenic potential of interactions and potential cancer-related signaling subnetworks. We envisage that the *graph2sig* itself and the weighted integrated network of human genes interactions, a network created in the first steps of *graph2sig*, will serve as platforms for elucidating the relationship between interactions and the expression of the malignant phenotype. Furthermore, as part of an integrative systems biology framework to facilitate the interpretation of cancer genome sequencing data [37], *graph2sig* could be used in two ways: the selection of the most relevant mutated genes according to their presence in high oncogenic interactions and the discovery of subnetworks most likely to be affected by these most relevant mutated gene. Finally, we also expect that the *graph2sig* can be used to predict and extract signaling pathways related to phenotypes other than cancer.

## Supporting Information

Figure S1
**The MET 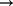 JUN subnetwork.** This subnetwork contains 116 interactions. The highlighted solid edges represent the interactions present in the corresponding OLP. The highlighted dashed edges represent the interactions of the potential oncogenic pathway (MET 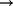 STAT3 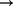 JUN). Blue edges represent protein physical interactions and orange nodes represent genes participating in the known or potential oncogenic pathways.(PDF)

Figure S2
**The KRAS 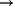 CCND1 subnetwork.** This subnetwork contains 134 interactions. The highlighted solid edges represent the interactions present in the corresponding OLP. The highlighted dashed edges represent the interactions of the potential oncogenic pathway (KRAS 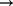 PIK3CA 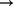 AKT1 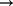 GSK3B 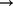 MYC 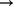 CCND1). Blue, red and green edges represent, respectively, protein physical, transcriptional regulation and metabolic interactions; orange nodes represent genes participating in the known or potential oncogenic pathways.(PDF)

Dataset S1 **Complete data for the** ***wINHGI.*** This is a tab-delimited file that includes a table containing all interactions of *wINGHI* with their type of interaction (protein physical, transcriptional regulation and metabolic interactions), calculated network centralities and oncogenic scores. Furthermore, it is also possible to find whether interactions are present in KEGG PATHWAY as oncogenic interactions (interactions used as positive examples in the training step) and whether interactions belong to the set of putative oncogenic interactions (oncogenic signal transduction events). The identification of interactors are EntrezGeneIDs. The list of interactions are ordered by oncogenic score.
(ZIP)

Dataset S2 **The 52 subnetworks constructed by** ***graph2sig.*** Tab-limited file containing all 52 subnetworks constructed by *graph2sig*. For each subnetwork are shown the interactions (the identification of interactors is the official gene symbol), the type of interaction (protein physical, transcriptional regulation and metabolic interactions) and the oncogenic scores.
(TXT)

Table S1 **Statistics for the 52 constructed subnetworks.** This spreadsheet shows the statistics for the 52 constructed subnetworks including the initial and target genes of the OLPs, the cancer type from which the OLPs were extracted, the sizes of OLPs and constructed subnetworks and the ratio between the number of interactions of OLPs in the subnetworks and the actual number of interactions in OLPs.
(PDF)
