# Supplementary figures and images for: Prediction of Oncogenic Interactions and Cancer-Related Signaling Networks Based on Network Topology
Source: PLoS One. 2013 Oct 25;8(10):e77521. doi: 10.1371/journal.pone.0077521 (PMC3808429; doi:10.1371/journal.pone.0077521)

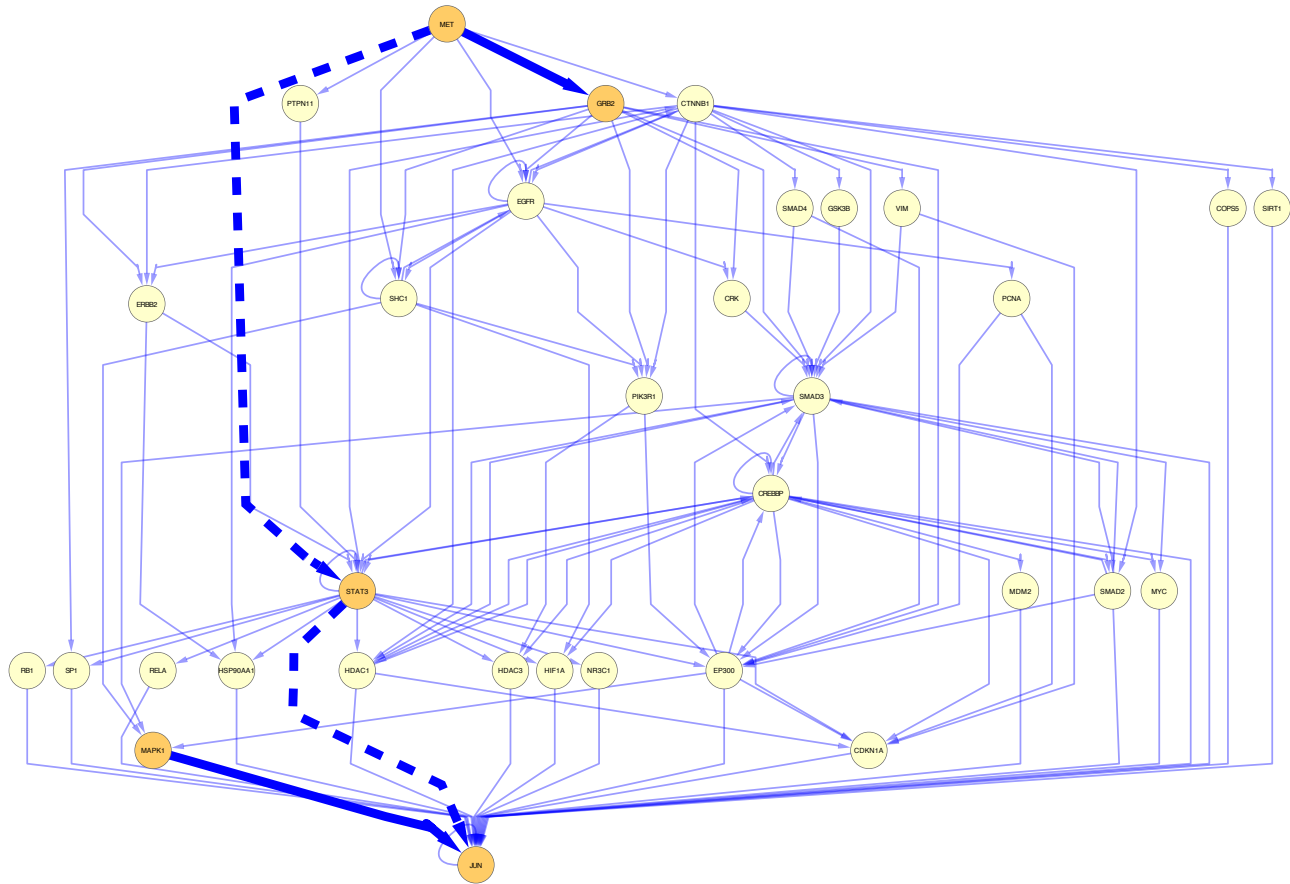

Supplement: Figure S1 — The MET JUN subnetwork. This subnetwork contains 116 interactions. The highlighted solid edges represent the interactions present in the corresponding OLP. The highlighted dashed edges represent the interactions of the potential oncogenic pathway (MET STAT3 JUN). Blue edges represent protein physical interactions and orange nodes represent genes participating in the known or potential oncogenic pathways. (PDF) [file pone.0077521.s001.pdf]

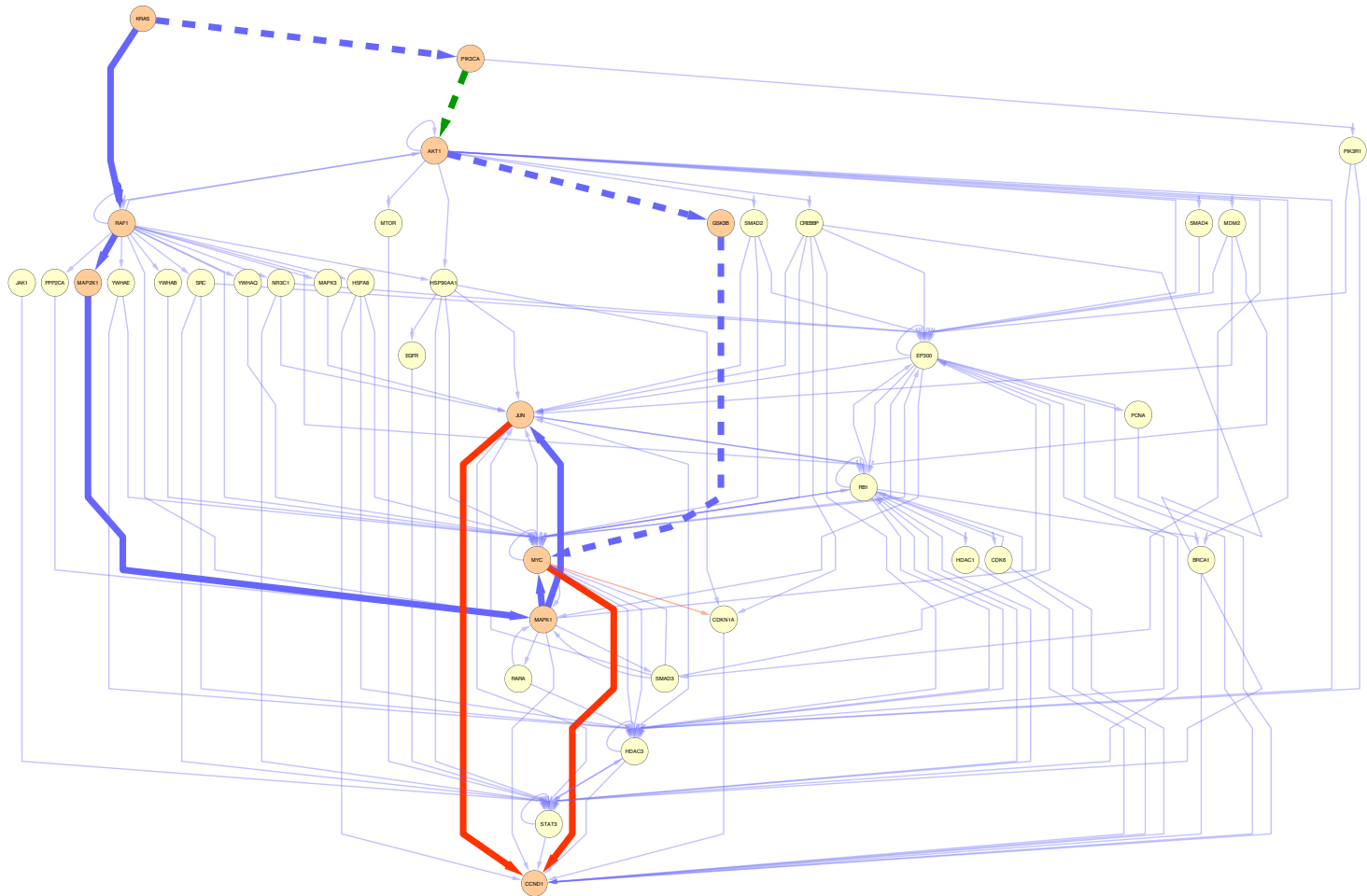

Supplement: Figure S2 — The KRAS CCND1 subnetwork. This subnetwork contains 134 interactions. The highlighted solid edges represent the interactions present in the corresponding OLP. The highlighted dashed edges represent the interactions of the potential oncogenic pathway (KRAS PIK3CA AKT1 GSK3B MYC CCND1). Blue, red and green edges represent, respectively, protein physical, transcriptional regulation and metabolic interactions; orange nodes represent genes participating in the known or potential oncogenic pathways. (PDF) [file pone.0077521.s002.pdf]
