# Supplementary material for: Prediction of Oncogenic Interactions and Cancer-Related Signaling Networks Based on Network Topology
Source: PLoS One. 2013 Oct 25;8(10):e77521. doi: 10.1371/journal.pone.0077521 (PMC3808429; doi:10.1371/journal.pone.0077521)
Supplement: Table S1 — Statistics for the 52 constructed subnetworks. This spreadsheet shows the statistics for the 52 constructed subnetworks including the initial and target genes of the OLPs, the cancer type from which the OLPs were extracted, the sizes of OLPs and constructed subnetworks and the ratio between the number of interactions of OLPs in the subnetworks and the actual number of interactions in OLPs. (PDF) [file pone.0077521.s005.pdf]

Oncogenic linear pathway (initial gene -> target gene)	Cancer type (KEGG Pathway code)	Oncogenic linear pathway size (number of interactions)	Subnetwork size (number of interactions)	Recall* (%)
PTEN -> TP53	Prostate cancer (hsa5215)	3	275	100
PTEN -> NFKB1	Prostate cancer (hsa5215)	4	411	100
PTEN -> LEF1	Prostate cancer (hsa5215)	4	2353	100
PDGFB -> MTOR	Glioma (hsa5214)	4	2652	100
NRAS -> MAPK3	Melanoma (hsa5218)	3	13	100
NRAS -> MAPK1	Melanoma (hsa5218)	3	164	100
KRAS -> RPS6KB1	Acute myeloid leukemia (hsa5221)	4	10	100
KRAS -> NFKB1	Pancreatic cancer (hsa5212)	4	389	100
KRAS -> IKBKB	Acute myeloid leukemia (hsa5221)	3	11	100
KRAS -> ELK1	Endometrial cancer (hsa5213)	4	577	100
KRAS -> EIF4EBP1	Acute myeloid leukemia (hsa5221)	4	15	100
KRAS -> CCND1	Colorectal cancer (hsa5210)	5	134	100
KRAS -> BCL2L1	Pancreatic cancer (hsa5212)	4	1203	100
KRAS -> BAD	Non-small cell lung cancer (hsa5223)	3	23	100
KIT -> RPS6KB1	Acute myeloid leukemia (hsa5221)	4	605	100
KIT -> MAPK3	Acute myeloid leukemia (hsa5221)	6	2686	100
KIT -> IKBKB	Acute myeloid leukemia (hsa5221)	3	1259	100
KIT -> EIF4EBP1	Acute myeloid leukemia (hsa5221)	4	3051	100
KIT -> BAD	Acute myeloid leukemia (hsa5221)	3	182	100
HRAS -> MAPK3	Bladder cancer (hsa5219)	3	3273	100
HRAS -> MAPK1	Bladder cancer (hsa5219)	3	35	100
FLT3 -> RPS6KB1	Acute myeloid leukemia (hsa5221)	4	678	100
FLT3 -> IKBKB	Acute myeloid leukemia (hsa5221)	3	1729	100
ERBB2 -> VEGFA	Pancreatic cancer (hsa5212)	3	24	100
EGFR -> MTOR	Glioma (hsa5214)	3	1202	100
CDKN2A -> E2F3	Glioma (hsa5214)	6	440	100
CDKN2A -> E2F2	Glioma (hsa5214)	6	885	100
CDKN2A -> E2F1	Glioma (hsa5214)	6	844	100
CDKN1B -> E2F3	Prostate cancer (hsa5215)	3	2888	100
CDKN1B -> E2F2	Prostate cancer (hsa5215)	3	487	100
CDKN1B -> E2F1	Prostate cancer (hsa5215)	3	306	100
ABL1 -> CDKN1B	Chronic myeloid leukemia (hsa5220)	4	1820	100
FLT3 -> MAPK3	Acute myeloid leukemia (hsa5221)	6	2199	83
ABL1 -> MAPK3	Chronic myeloid leukemia (hsa5220)	5	1099	80
PDGFB -> MAPK3	Glioma (hsa5214)	8	1548	75
PDGFB -> MAPK1	Glioma (hsa5214)	8	759	75
PDGFA -> MAPK3	Glioma (hsa5214)	8	2203	75
FLT3 -> EIF4EBP1	Acute myeloid leukemia (hsa5221)	4	169	75
EGFR -> MAPK3	Glioma (hsa5214)	7	3005	71
PDGFA -> MTOR	Glioma (hsa5214)	4	17	50
KRAS -> MYC	Colorectal cancer (hsa5210)	4	10	50
FLT3 -> BAD	Acute myeloid leukemia (hsa5221)	7	12	43
PTEN -> CCND1	Small cell lung cancer (hsa5222)	5	473	40

PTEN -> BIRC3	Small cell lung cancer (hsa5222)	5	500	40
MET -> JUN	Renal cell carcinoma (hsa5211)	7	116	29
EGFR -> MAPK1	Glioma (hsa5214)	7	988	29
ABL1 -> TP53	Chronic myeloid leukemia (hsa5220)	5	17	20
ABL1 -> MAPK1	Chronic myeloid leukemia (hsa5220)	5	20	20
KIT -> MAPK1	Acute myeloid leukemia (hsa5221)	6	48	17
FLT3 -> MAPK1	Acute myeloid leukemia (hsa5221)	6	30	17
ABL1 -> NFKB1	Chronic myeloid leukemia (hsa5220)	6	18	17
PDGFA -> MAPK1	Glioma (hsa5214)	8	35	13

* Ratio between the number of interactions of the OLP in the subnetwork and the actual number of interactions in OLP

(green)	Recall = 100%
(yellow)	100% > Recall > 49%
(orange)	50% > Recall > 25%
(red)	Recall < 25%
